# Differential Roles of PML Isoforms

**DOI:** 10.3389/fonc.2013.00125

**Published:** 2013-05-22

**Authors:** Sébastien Nisole, Mohamed Ali Maroui, Xavier H. Mascle, Muriel Aubry, Mounira K. Chelbi-Alix

**Affiliations:** ^1^INSERM UMR-S 747Paris, France; ^2^Université Paris DescartesParis, France; ^3^CNRS FRE3235Paris, France; ^4^Département de Biochimie, Université de MontréalMontréal, QC, Canada

**Keywords:** PML nomenclature, PML isoforms, TRIM, SUMO, SIM, RNF4, CK2, As_2_O_3_, virus

## Abstract

The tumor suppressor promyelocytic leukemia (PML) protein is fused to the retinoic acid receptor alpha in patients suffering from acute promyelocytic leukemia (APL). Treatment of APL patients with arsenic trioxide (As_2_O_3_) reverses the disease phenotype by a process involving the degradation of the fusion protein *via* its PML moiety. Several PML isoforms are generated from a single *PML* gene by alternative splicing. They share the same N-terminal region containing the RBCC/tripartite motif but differ in their C-terminal sequences. Recent studies of all the PML isoforms reveal the specific functions of each. Here, we review the nomenclature and structural organization of the PML isoforms in order to clarify the various designations and classifications found in different databases. The functions of the PML isoforms and their differential roles in antiviral defense also are reviewed. Finally, the key players involved in the degradation of the PML isoforms in response to As_2_O_3_ or other inducers are discussed.

## Introduction

The promyelocytic leukemia (*PML*) gene was originally identified in acute promyelocytic leukemia (APL) where it is fused to the retinoic acid receptor alpha (*RARA*) gene as a result of a t(15; 17) chromosomal translocation (de The et al., 1991; Kakizuka et al., 1991; Pandolfi et al., 1991). This translocation leads to the synthesis of a chimeric protein, named PML-RARα, which blocks the differentiation of hematopoietic progenitor cells. In normal cells, PML forms nuclear speckles, known as PML nuclear bodies (NBs). In APL cells, due to the expression of PML-RARα (Dyck et al., 1994; Weis et al., 1994), NBs are dispersed as microspeckles. Thus, alteration of PML NB functions by PML-RARα expression may contribute to leukemogenesis. The treatment of APL patients with arsenic trioxide (As_2_O_3_) reverses the disease phenotype and cures up to 70% of APL patients (Mathews et al., 2010). Remarkably, by targeting the PML moiety, As_2_O_3_ promotes PML-RARα degradation which leads to PML NB reformation (Zhu et al., 1997). Also, As_2_O_3_ increases PML SUMOylation and promotes its interaction with the poly-SUMO-dependent ubiquitin E3 ligase that is responsible for proteasome-mediated PML degradation, namely RNF4 for Really interesting New gene (RING) Finger protein 4 (Lallemand-Breitenbach et al., 2008; Tatham et al., 2008).

Both the covalent conjugation of SUMO to PML and the non-covalent interaction of SUMO with the SUMO Interacting Motif (SIM) of PML are required for the integrity and normal function of PML NBs (Ishov et al., 1999; Shen et al., 2006). PML NBs are dynamic structures, which harbor a few permanently (PML, Sp100, and SUMO) and numerous transiently residing proteins depending on different conditions (i.e., transformation, stress, interferon (IFN) treatment, and viral infections). The list of cellular and viral proteins recruited on PML NBs, based on co-localization studies, is growing. Interestingly, based on database analyses, 166 proteins were found to be associated with PML (Van Damme et al., 2010). Since the discovery of PML in APL, numerous studies have been conducted to link PML and PML NBs with various cellular functions including senescence, apoptosis, protein degradation, and antiviral defense (Everett and Chelbi-Alix, 2007; Bernardi et al., 2008; Krieghoff-Henning and Hofmann, 2008; Geoffroy and Chelbi-Alix, 2011; Rabellino and Scaglioni, 2013). In fact, the interaction of PML with certain viral proteins or with the PML-RARα chimeric protein causes PML NB disruption and abolishes normal PML NB functions.

Promyelocytic leukemia is the organizer of the NBs. Several PML isoforms, designated PMLI to PMLVIIb, which differ within their C-terminal end, are expressed by alternative splicing of a single *PML* gene. The variations in the COOH-terminal regions lead to the specific functions of each PML isoform. For example, in the context of antiviral defense, during varicella-zoster virus (VZV) (Reichelt et al., 2011) or encephalomyocarditis virus (EMCV) (Maroui et al., 2011) infection, only PMLIV sequesters viral proteins in PML NBs and inhibits viral production. In recent years, although few comparative studies have been performed with all the PML isoforms, increasing evidence suggests that each PML isoform exhibits specific functions.

In this review, we will discuss first the nomenclature and structural organization of the PML isoforms in order to clarify the classifications that are found in different databases. Then, we will review the role of key players in the As_2_O_3_-induced degradation of PML isoforms as compared to other inducers. Also, we will analyze how a particular PML isoform confers viral resistance by sequestering viral proteins in PML NBs and the strategies developed by these viruses to disrupt these structures. Finally, we will summarize the functions attributed to each isoform.

## Nomenclature and Structure of the PML Isoforms

### Nomenclature for the PML isoforms

**Figure 1:**
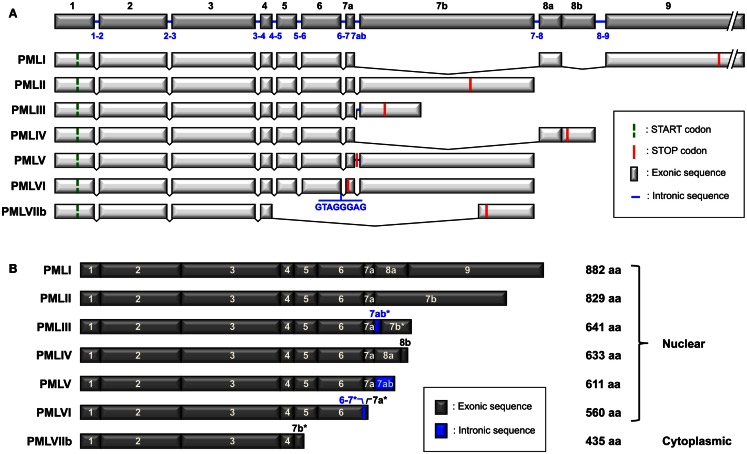
**Structure of the *PML* gene and the PML isoforms generated by alternative splicing**. **(A)** *PML* gene (NCBI accession no. NG_029036.1) includes nine exons (1–9) according to the nomenclature described by Jensen et al. (2001). Exons 7 and 8 can be subdivided into exons 7a, 7b, 8a, and 8b. Note that the 7ab sequence corresponds to the retained intronic region between exon 7a and 7b found in PMLIII and PMLV. There are some differences for exons 6, 7, and 8 between the nomenclature of Jensen and that of the NCBI (see accession numbers in Table 1). Exon 6 in Jensen’s nomenclature corresponds to exon 6a in NCBI, exon 7b of PMLIII to exon 7c, exons 7a-7ab-7b to exon 7b, exon 7b of PMLVIIb to exon 7d, exons 8a-8b to exon 8b. Alternative splicing of the *PML* gene leads to seven main mRNA variants. The start and stop codons are symbolized for each variant. The retained intronic sequences in PMLIII and PMLV (between exons 7a and 7b), as well as in PMLVI (between exons 6 and 7a) are indicated. Retained introns introduce a frameshift in exon 7b of PMLIII and in exon 7a of PMLVI. Only exons are represented at the same scale. Intron length: 1–2: 3,062 bp/2–3: 24,351 bp/3–4: 1,448 bp/4–5: 7,644 bp/5–6: 440 bp/6–7: 1,063 bp/7ab: 640 bp/7–8: 6,594 bp/8–9: 844 bp. **(B)** The length of the main PML isoforms (PMLI to PMLVIIb) encoded by the different mRNA variants and their exon composition, from the initiating methionine to the end of the protein, are shown. The asterisks indicate that only part of the sequence (either exon or intron) is retained (due to the presence of an in frame STOP codon).

**Figure 2:**
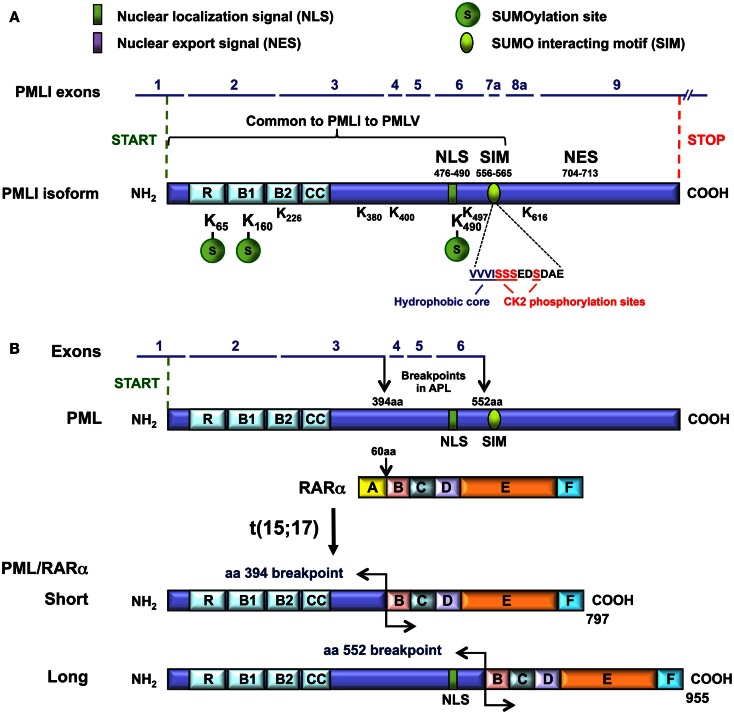
**Domain organization of the PML and the PML-RARα proteins**. **(A)** The domains, the motifs, and their amino acid (aa) positions relative to the PMLI isoform are presented. These include the RBCC/TRIM, the NLS, the NES, and the SIM hydrophobic core (VVVI) motifs as well as the adjacent sequences containing the CK2-phosphorylation sites. The three major (K65, K160, and K490) and five minor (K226, 380, 400, 497, 616) SUMOylation sites are shown. A bracket indicates the common region shared by PMLI to PMLV and encoded by exons 1 to 7a. The SIM encoded by exon 7a is missing in PMLVI and PMLVIIb. The K616 encoded by exon 8a is found only in PMLI and PMLIV. The NES is present only in PMLI. **(B)** The short and the long PML-RARα fusion proteins and their translocation breakpoints are shown.

Promyelocytic leukemia isoforms are generated by alternative splicing from a single *PML* gene, which includes nine exons according to the original nomenclature defined by Jensen et al. (2001) (Figure 1A). Exons 7 and 8 can be divided into exons 7a, 7b, 8a, and 8b. Some isoforms also contain the 7ab intronic sequence in whole (7ab) or in part (7ab*) (Figure 1B). There are six nuclear PML isoforms designated PMLI to PMLVI and one cytoplasmic isoform, PMLVIIb (called PMLVII in some publications) (Figure 1B) (Jensen et al., 2001). The isoforms are numbered in the inverse order of their size, PMLI being the longest and PMLVIIb the shortest. They share the same N-terminal region but have different C-termini due to alternative splicing of exons 4–9 (Figure 1). In addition to the seven main isoforms (PMLI to PMLVIIb), other potential PML isoforms might be expressed as a result of putative alternative splicing of exons 4, 5, and 6. Letters are added to the names of the isoforms to indicate the lack of particular exons; “a” is for isoforms without exon 5 (amino acids 419–466), “b” for isoforms without exon 5 and 6 (amino acids 419–552) and “c” for isoforms without exon 4, 5, and 6 (amino acids 395–552). For example, PMLIVa corresponds to PMLIV isoform without exon 5 and PMLVIb corresponds to PMLVI without exon 5 and exon 6. Since the “b” and “c” variants do not have the nuclear localization signal (NLS) encoded by exon 6 (Figure 2A), they are likely to be cytoplasmic as is the case for PMLVIIb. Note that in addition to the loss of exons 5 and 6, exon 7a, which is found in PMLI to PMLVI, is also missing in PMLVIIb.

Interestingly, the splicing of exons 5 and 6 in the mRNA variants PMLI to PMLVI (which retain exon 7a) results in a frameshift which places a STOP codon at the beginning of exon 7a and, thus, leads to transcripts coding for a single cytoplasmic protein cPMLΔ5-6 (corresponding to PMLIb to PMLVIb). Note that the eight nucleotide intronic sequence within PMLVI is located at the end of exon 6. This short sequence is not present after the splicing of exons 5–6. Therefore, the PMLVIb protein will be identical to the other “b” variants. cPMLΔ5-6 (423 amino acids) and PMLVIIb (435 amino acids) differ from each other by only 12 amino acids at their C-terminal end. The C-terminus of cPMLΔ5-6 (RNALW) is encoded by the very beginning of exon 7a (identical to the C-terminus of PMLVI), whereas the C-terminus of PMLVIIb (VPPPAHALTGPAQSSTH) is encoded, out of frame, by a part of exon 7b. In contrast, the splicing of exons 4, 5, and 6 results in transcripts which code for cytoplasmic proteins which contain the specific C-terminal region of each isoform (PMLIc to PMLVIc). In the case of exon 5 splicing, the transcripts encode nuclear PML isoforms, which contain the corresponding C-terminal region of each (PMLIa to PMLVIa). Further studies are needed to confirm the suggestion of Fagioli et al. (1992) that all isoforms can be expressed endogenously from a, b, or c mRNA variants.

This nomenclature (Jensen et al., 2001) differs from other classifications provided by NCBI, GenBank, and UniProt (Table 1). The Jensen nomenclature is usually used by researchers in the PML field for its convenience and its coherence relative to the organization of the *PML* gene, the mRNA spliced variants, and the resulting protein isoforms. For further clarification, we provide a compilation of the various names given to PML isoforms in these classification systems as well as their accession numbers and length (Table 1). Since the names given to PML isoforms within different databases conflict, the systematic inclusion in publications of the accession number would permit the proper identification of the PML isoforms used. Of note, the name of some exons differs between the classification originally used (Jensen et al., 2001) and the NCBI database (see Figure 1A legend). For example, the exons 8a–8b in PMLIV correspond to NCBI exon 8b (Figure 1A). Finally, a few of the listed isoforms correspond to polymorphic sequences with different numbers of tandem repeats (e.g., PMLII isoforms with two or three repeats of the pentapeptide SSPAH leading to proteins with 824 or 829 amino acids, respectively) (Table 1).

**Table 1:** **Nomenclature of the PML isoforms**.

Jensen et al. name	Isoform length	TRIM name	GenBank name	GenBank accession number	NCBI name	NCBI accession number	UniProt name	UniProt accession number
PMLI	882 aa	TRIM19 alpha	TRIM19 alpha	AF230401	Isoform 1	NP_150241.2	PML-1^1^	P29590-1
	860 aa^2^		PML-1^1^	M79462				
PMLIa^5^	834 aa						PML-11	P29590-11
PMLII	854 aa^3^^,^^4^	TRIM19 delta	TRIM19 delta	AF230404				
	829 aa	TRIM19 kappa	TRIM19 kappa	AF230410	Isoform 9	NP_150242.1	PML-2^1^	P29590-8
	824 aa^4^	TRIM19 gamma	TRIM19 gamma	AF230403			PML-8	P29590-3
	802 aa^2^^,^^4^		PML-3^1^	M79464				
PMLIIa^5^	781 aa				Isoform 11	NP_150253.2	PML-13	P29590-13
PMLIII	641 aa		PML	S50913			PML-3^1^	P29590-9
PMLIV	633 aa	TRIM19 zeta	TRIM19 zeta	AF230406	Isoform 6	NP_002666.1	PML-4	P29590-5
	633 aa		Myl (PML)	X63131				
PMLIVa^5^	585 aa	TRIM19 lambda	TRIM19 lambda	AF230411	Isoform 10	NP_150252.1	PML-12	P29590-12
PMLV	611 aa	TRIM19 beta	TRIM19 beta	AF230402	Isoform 2	NP_150243.2	PML-5	P29590-2
	589 aa^2^		PML-2^1^	M79463				
PMLVI	560 aa	TRIM19 epsilon	TRIM19 epsilon	AF230405	Isoform 5	NP_150247.2	PML-6	P29590-4
	560 aa		PML-1^1^	M73778				
	538 aa^2^		PML-3B	M80185				
PMLVIb^6^	423 aa	TRIM19 iota	TRIM19 iota	AF230409	Isoform 7	NP_150249.1	PML-14	P29590-14
	423 aa	TRIM19 eta	TRIM19 eta	AF230407				
PMLVIIb^7^	435 aa	TRIM19 theta	TRIM19 theta	AF230408	Isoform 8	NP_150250.2	PML-7	P29590-10

*^1^Conflicting name given for different isoforms in GenBank and UniProt*.

*^2^Protein product reported to start at Met23 in GenBank even if the mRNA sequence contains the sequence coding for the first 22 amino acids (aa) including the Met1*.

*^3^Same sequence as for AF230403 (824 aa) but which contains an extra 30 aa repeat in exon 7b (repeated sequence: QRGISPPHRIRGAVRSRSRSLRGSSHLSQW)*.

*^4^Two repeats of the pentapeptide SSPAH instead of three in exon 7b in PMLII*.

*^5^Isoform missing exon 5 (aa 416–466)*.

*^6^Isoform missing exons 5 and 6 (aa 416–552) that is identical to cPMLΔ5-6 (see Table 5). Note that, as described in Jensen et al. (2001) and reported by the NCBI and UniProt databases, this isoform is missing the intron sequence 5^′^-GTAGGGAG-3^′^ (shown in Figure 1A) present in PMLVI at the end of exon 6*.

*^7^Due to the absence of exons 5 and 6, this isoform is referred as PMLVIIb instead of PMLVII*.

### Domain structure of PML isoforms

Promyelocytic leukemia is a member of the tripartite motif (TRIM) family (Reymond et al., 2001; Nisole et al., 2005). The RBCC/TRIM motif (at amino acids 57–253 in exons 1–3) harbors a C_3_HC_4_ RING-finger, two B-boxes (B1 and B2), and an α-helical coiled-coil domain (Figure 2A) (Kastner et al., 1992). As a member of the TRIM family, PML corresponds to TRIM19. Specific names have been given to the PML isoforms according to the TRIM classification (Table 1).

All the PML isoforms share exons 1–3, which encode the RBCC/TRIM motif at their N-terminal end (Figure 2A). This motif is essential for PML NB formation and PML homodimerization *via* the coiled–coiled domain. The differences in the C-terminal parts of the PML isoforms (Figure 1B) determine the different partners and, thus, the specific functions of each isoform. Several motifs have been identified in the C-termini of PML isoforms (Figure 2A). The nuclear export signal (NES) (amino acids 704–713/exon 9) is found only in PMLI, consistent with the nuclear and cytoplasmic distribution of this isoform. The SIM is present only in PMLI to PMLV. The SIM hydrophobic core (VVVI encoded by exon 7a, amino acids 556–559) is adjacent to specific serines (S560, S561, S562, and S565) which are substrates for the Casein Kinase-2 (CK2) (Scaglioni et al., 2006; Shen et al., 2006). Note that these two studies used the PML isoform PMLIVa, which lacks exon 5, where the SIM hydrophobic core is located at amino acids 508–511 and the CK2 phosphorylated sites at positions S512, S513, S514, and S517. Both the SIM hydrophobic core and the CK2-phosphorylation sites are missing in the nuclear PMLVI (due to a short retained intron at the end of exon 6 which introduces a frameshift in exon 7a) and in the cytoplasmic PMLVIIb (absence of exon 7a) (Figure 1).

### Domain structure of PML-RARα

In the t(15; 17) translocations of APL, two major breakpoints have been identified in the *PML* gene that is fused to *RARA* (de The et al., 1991; Pandolfi et al., 1991; Kastner et al., 1992). The *RARA* breakpoint invariably occurs within the second intron of this gene. The breakpoints in *PML* are located between exons 3 and 4 (at amino acid 394) and between exons 6 and 7 (at amino acid 552) and lead to two PML-RARα fusion proteins, S for Short fusion and L for Long fusion (Figure 2B). The PML-RARα L isoform is expressed in approximately 55% of adult patients with APL, whereas the S isoform is expressed in approximately 35% of the patients (Zelent et al., 2001). In addition, another chimeric protein in APL can be generated by the alternative splicing of exon 5 in PML-RARα transcripts (de The et al., 1991; Kastner et al., 1992). All the PML-RARα fusion proteins possess the RBCC motif but lack the various C-terminal regions, which are characteristic of each PML isoform including the SIM and CK2 phosphorylating sites (in exon 7a). Although the short PML-RARα isoform differs from the long one since it lacks the PML NLS (in exon 6) and some of the SUMOylation sites in exons 4 and 6, it maintains a nuclear localization due to its RARα moiety.

The t(15; 17) translocation disrupts one allele of the *PML* gene and, consequently, reduces PML mRNA expression. The formation of heterodimers between PML and PML/RARα *via* the coiled-coil domain facilitates the sequestration of PML out of the NBs. In APL, the chimeric PML-RARα protein alters the normal localization of PML NBs from the speckled pattern to a pattern of micro-dispersed tiny dots. All the PML NB-associated partners are delocalized in APL cells. The disruption of PML and RARα functions are implicated in APL pathogenicity because PML-RARα impairs both nuclear receptor-induced differentiation and PML-triggered apoptosis (Nason-Burchenal et al., 1998; Mistry et al., 2003). Thus, inactivation of the PML growth suppressor activity may result in uncontrolled growth of APL cells.

## Key Steps in As_2_O_3_-Induced Degradation of PML Isoforms

**Figure 3:**
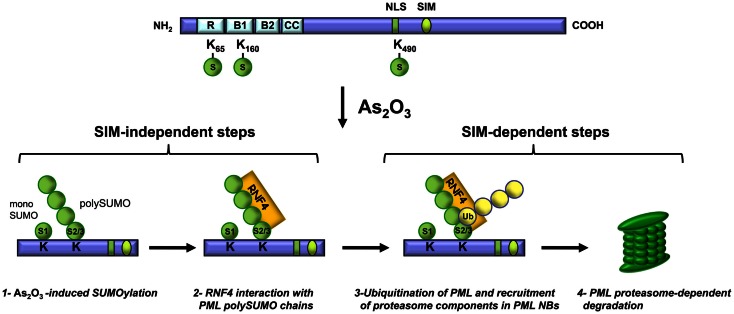
**Key steps in As_2_O_3_-induced PML degradation**. Steps 1 and 2 are SIM-independent whereas steps 3 and 4 require the PML SIM.

Since the discovery that As_2_O_3_ reverses the disease phenotype of APL patients, numerous studies have been conducted to elucidate the mechanisms of action of this therapeutic agent in normal and cancer cells. As_2_O_3_ was shown to promote PML and PML-RARα degradation. Briefly, before As_2_O_3_ induces degradation, PML is phosphorylated, is transferred from the nucleoplasm to the nuclear matrix, is SUMOylated and it interacts, *via* the SUMO moiety, with RNF4. These steps are followed by PML ubiquitination and the recruitment of proteasome components to PML NBs, which results in proteasome-dependent degradation of PML (Figure 3). These findings, together with others, establish that PML NBs are sites of protein modification and degradation (Fogal et al., 2000; Lallemand-Breitenbach et al., 2001, 2008; Pampin et al., 2006; Tatham et al., 2008; Malloy et al., 2013).

### As_2_O_3_ induces the transfer of PML to the nuclear matrix-associated NBs

In the nucleus, PML is expressed mostly in the diffuse nuclear fraction of the nucleoplasm (RIPA soluble fraction) while a small fraction is found in the matrix-associated NBs (RIPA resistant fraction) (Zhu et al., 1997; Muller et al., 1998; Porta et al., 2005; Pampin et al., 2006; El Mchichi et al., 2010). PML is detected mainly as the bright immunofluorescent speckles, which form the NBs even though PML is more abundant in the nucleoplasm. In response to As_2_O_3_, PML is phosphorylated rapidly (within 10 min) in its N-terminus (T28, S36, S38, and S40) by ERK1/2 mitogen-activated protein kinase (Hayakawa and Privalsky, 2004) and, then, transferred from the nucleoplasm to the nuclear matrix (Lallemand-Breitenbach et al., 2001). Interestingly, this transfer occurs independently of PML SUMOylation as a SUMOylation-deficient mutant (PMLIII-3KR) still is transferred to the nuclear matrix in cells treated with As_2_O_3_ (Lallemand-Breitenbach et al., 2001). Note that poliovirus infection also very rapidly induces PML phosphorylation by the ERK1/2 pathway, followed by the transfer of PML from the nucleoplasm to the nuclear matrix (Pampin et al., 2006). However, further experiments using PML phosphorylation-deficient mutants are needed to demonstrate that PML phosphorylation by ERK1/2 is sufficient to induce the transfer of PML toward the nuclear matrix.

### As_2_O_3_ enhances PML SUMOylation

#### PML SUMOylation and PML NB formation

Promyelocytic leukemia NBs provide a nuclear platform for post-translational modifications such as phosphorylation, acetylation, and SUMOylation (Cheng and Kao, 2012; Schmitz and Grishina, 2012). SUMOylation is the most studied post-translational modification of PML. SUMOylation is believed to occur in the nuclear matrix and it has important consequences for PML functions because it can affect the localization, stability, and ability of PML to interact with other partners. SUMOylation of PML is critical for NB formation since PML SUMO-deficient mutants are unable to form these structures (Ishov et al., 1999). The requirement for a functional SUMO machinery for PML NB formation also was demonstrated in cells derived from knockout mice for SUMO1 or the E2 SUMO-conjugating enzyme, UBC9 (Nacerddine et al., 2005; Evdokimov et al., 2008). Recently, it has been reported that the SUMO E3 ligase PIAS1, for Protein Inhibitor of Activated STAT, interacts with the PML RBCC motif and enhances PML SUMOylation (Rabellino et al., 2012).

Modification by SUMO is a common property of PML NB-associated proteins. According to the current literature, almost 40% of PML partners have been confirmed to be SUMOylated. This suggests that PML NBs are enriched sites for SUMOylated proteins and may function as nuclear SUMOylation hotspots (Van Damme et al., 2010).

#### SUMOylation sites in PML

Initial studies identified three lysine residues (K65, K160, K490 located, respectively, in the RING-finger, B1 box, and NLS) in PML as major SUMOylation sites. These lysine residues are all part of the canonical SUMOylation consensus motif ψKxE/D or its inverted version E/DKxψ (Kamitani et al., 1998; Da Silva-Ferrada et al., 2012) (Table 2). PML is modified by covalent coupling of SUMO1, SUMO2, and SUMO3 to these three lysine residues (K65, K160, K490) (Kamitani et al., 1998). However, residual SUMOylation of PMLIII-3KR (mutated in K65, K160, and K490) still is detected in the presence of overexpressed SUMO and is enhanced upon overexpression of PIAS1 or As_2_O_3_ treatment (Galisson et al., 2011; Rabellino et al., 2012). This indicates that PML still can be SUMOylated even in the absence of the three major SUMOylation sites, although the extent of this modification is significantly lower than that observed with PMLIII wild-type. These observations are consistent with the identification of five additional SUMOylation sites (K226, K380, K497, K400, K616) which correspond to potential minor SUMOylation sites, three of which are compliant with the canonical consensus or its inverted version (K226, K380, and K616) (Vertegaal et al., 2006; Galisson et al., 2011) (Table 2). These SUMOylation sites are present in the nuclear PMLI to PMLVI, except K616 encoded by exon 8a that is only found in PMLI and PMLIV. Note that residues K380 and K400 were identified previously as sites of polyubiquitination in response to As_2_O_3_ (Tatham et al., 2008). Futures studies will reveal the potential role of these new SUMOylation sites in PML functions.

**Table 2:** **Major and minor PML SUMOylation sites**.

Consensus		PML SUMOylation sites		Exon	Motif	Reference
CM	Ψ 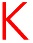 xE/D	K226: L 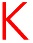 CD	Minor	3	B2	Vertegaal et al. (2006)
		K616: L 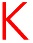 ID	Minor	8a	–	
ICM	E/Dx 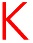 Ψ	K65: EA 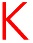 C	Major	2	RING	Kamitani et al. (1998)
		K380: EF 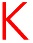 V	Minor	3	–	Galisson et al. (2011)
HCSM	ΨΨΨ 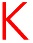 xE	K160: WLF 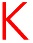 HE	Major	2	B1	Kamitani et al. (1998)
NDSM	Ψ 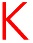 xExxEEE	K490: I 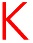 MESEE	Major	6	NLS	
Other		K400: VS 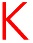 KASP	Minor	4	–	Galisson et al. (2011)
		K497: EEG 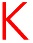 EAR	Minor	6	–	

*The longest PML isoform, PMLI, contains 30 lysines and those targeted by SUMO are numbered in reference to this isoform. Most identified PML SUMOylation sites identified correspond to known SUMO Consensus Motif (CM) and its variants (Da Silva-Ferrada et al., 2012). These include the inverted version of the CM (ICM: *I*nverted *C*onsensus *M*otif) and variants of the CM with extensions that favor SUMOylation such as an amino-terminal hydrophobic extension (HCSM: *H*ydrophobic *C*luster *S*UMO *M*otif) or a carboxy-terminal acidic extension (NDSM: *N*egatively charged amino-acid-*D*ependent *S*UMO *M*otif). Hydrophobic residue (Ψ), any amino acid (x), SUMOylation target lysine (in red)*.

#### As_2_O_3_ enhances poly-SUMOylation of nuclear PML isoforms

Except for the cytoplasmic PMLVIIb, all the PML isoforms (PMLI to PMLVI) are SUMOylation substrates (Maroui et al., 2012). PML SUMOylation is enhanced by various stimuli including As_2_O_3_ treatment and viral infections (Muller et al., 1998; Lallemand-Breitenbach et al., 2001; Pampin et al., 2006; El Mchichi et al., 2010). This post-translational modification is associated with the recruitment of PML NB partners and an increase in PML NB size. Interestingly, the inhibition of ERK1/2 with the MEK1 inhibitor U0126 abrogates both As_2_O_3_- and poliovirus-induced PML SUMOylation (Hayakawa and Privalsky, 2004; Pampin et al., 2006).

It has been suggested that As_2_O_3_ binds directly to cysteine residues in the zinc finger motifs located within the RBCC domain of PML leading to PML oligomerization and increased interaction of PML with the SUMO-conjugating enzyme UBC9 (Zhang et al., 2010). Furthermore, due to its oxidative properties, As_2_O_3_ also stimulates the oligomerization of PML by enhancing PML intermolecular disulfide bond formation, a process, which favors PML NB formation (Jeanne et al., 2010). Subsequently, As_2_O_3_ stimulates the formation of higher molecular weight poly-SUMO chains on all nuclear PML isoforms (Maroui et al., 2012).

### As_2_O_3_ promotes the interaction of all SUMOylated PML isoforms with RNF4

RNF4 is a SUMO targeted ubiquitin ligase (STUbL), which harbors a RING domain that strongly interacts with poly-SUMOylated PML owing to its multiple SIMs (Lallemand-Breitenbach et al., 2008; Tatham et al., 2008). These interactions occur with all nuclear SUMOylated PML isoforms (PMLI to PMLVI) and are increased in response to As_2_O_3_ (Maroui et al., 2012). These observations are consistent with the higher affinity of RNF4 for polySUMO chains and the strong induction of PML poly-SUMOylation elicited by As_2_O_3_ (Lallemand-Breitenbach et al., 2008; Tatham et al., 2008). The interaction of RNF4 with PML involves indirect binding *via* the SUMO moiety of PML-SUMO conjugates since no interaction was detected with a SUMOylation-deficient PML mutant (PMLIII-3KR) (Percherancier et al., 2009).

As_2_O_3_ is known to induce proteasomal degradation of PML (Zhu et al., 1997). RNF4 is a key player in PML proteasomal degradation since it acts as a poly-SUMO-specific E3 ubiquitin ligase that triggers the ubiquitination of poly-SUMOylated PML (Lallemand-Breitenbach et al., 2008; Tatham et al., 2008). As suggested in these studies, the ubiquitination can occur on either moiety of the PML-SUMO conjugates. Very recently, while this review was under revision, it was reported that Arkadia also acts as a poly-SUMO-targeted ubiquitin ligase implicated in As_2_O_3_-induced PML degradation (Erker et al., 2013) (see below). This finding sheds a new light on the complexity of the mechanisms of PML degradation in response to As_2_O_3_.

### PML SIM is required for As_2_O_3_-induced PML ubiquitination and degradation

SUMO proteins, as other ubiquitin-like proteins, not only can be conjugated covalently to proteins but also can form non-covalent interactions with various proteins containing a SIM (Song et al., 2004). The SIM (also named SBD for SUMO Binding Domain) identified in PML (Shen et al., 2006) is encoded by exon 7a, which is only translated in PMLI to PMLV. The hydrophobic core (VVVI) of the PML SIM is the minimal requirement for an interaction with SUMO and adjacent acidic and serine residues (SSSEDSDAE) follow this core (Figure 2). The serine residues are targets for CK2-phosphorylation (Scaglioni et al., 2006). The negative charges intrinsic to acidic residues or introduced by phosphorylation favor the interaction between SIMs and SUMO (Stehmeier and Muller, 2009).

The SIM of PML is not essential for NB formation as PMLVI, which lacks the SIM, still is able to form NBs when expressed in PML^−/−^ cells (Brand et al., 2010). However, the PML SIM hydrophobic core (VVVI) mediates non-covalent interactions with SUMOylated proteins and promotes their recruitment into PML NBs (Shen et al., 2006). In addition, the CK2-phosphorylation sites (*SSS*ED*S*DAE) are required for the positive regulation of this process, suggesting the phospho-dependent recruitment of some SUMOylated proteins (Percherancier et al., 2009). Therefore, both the SIM hydrophobic core and the adjacent CK2-phosphorylation sites are involved in the integrity and the normal function of PML NBs. Furthermore, the SIM is not required for PML SUMOylation and interaction with RNF4 as shown by results obtained with PMLVI which lacks the SIM or with a PMLIII-SIM_VVVI_ mutant (VVVI mutated to AAAS) (Percherancier et al., 2009; Maroui et al., 2012). In contrast, the SIM hydrophobic core is implicated in PML degradation since PMLVI or a PMLIII-SIM_VVVI_ mutant are not degraded efficiently upon As_2_O_3_ treatment of cells (Percherancier et al., 2009; Maroui et al., 2012) (Figure 3). The resistance of PMLVI or a PMLIII-SIM_VVVI_ mutant to the degradation process is due to their inability to be polyubiquitinated and to efficiently recruit the proteasome components to PML NBs (Maroui et al., 2012). Thus, not all PML nuclear isoforms are efficiently degraded in response to As_2_O_3_. Remarkably, PMLVI resistance to As_2_O_3_-induced degradation is bypassed by overexpression of RNF4 (Maroui et al., 2012).

Taken together, these results demonstrate that the SIM hydrophobic core is required for efficient As_2_O_3_-induced PML degradation, an event that occurs after the recruitment of RNF4 by poly-SUMOylated PML.

### As_2_O_3_ is a therapeutic agent of APL patients

Treatment of APL patients with *all-trans* retinoic acid (ATRA) or As_2_O_3_, reverses the disease phenotype and promotes PML-RARα degradation, which leads to PML NB reformation (Zhu et al., 1997, 1999). ATRA and As_2_O_3_ degrade the fusion protein by targeting RARα and PML, respectively, by two different mechanisms. APL was first shown to be sensitive to the differentiation therapy with ATRA (Huang et al., 1988). However some patients became resistant to treatment with ATRA. Interestingly, As_2_O_3_ is beneficial even in APL patients who have relapsed. The action of As_2_O_3_ in APL occurs through the induction of apoptosis and partial differentiation (Chen et al., 1997).

Promyelocytic leukemia-RARα is, like PML, phosphorylated by ERK1/2 in response to As_2_O_3_ (Hayakawa and Privalsky, 2004) and, then, highly conjugated to SUMO2/3 (Lallemand-Breitenbach et al., 2008), since it retains two of the three major PML SUMOylation sites at K65 and K160. As_2_O_3_ causes the poly-SUMOylation of PML-RARα and thus promotes its interaction with RNF4 leading to its proteasome-dependent degradation (Lallemand-Breitenbach et al., 2008; Tatham et al., 2008; Weisshaar et al., 2008). Thus, RNF4-mediated PML-RARα ubiquitination and degradation plays an important role in the APL therapeutic response to As_2_O_3_.

Surprisingly, the SIM hydrophobic core that is required for As_2_O_3_-induced RNF4-dependent degradation and the adjacent CK2-phosphorylation sites are missing in the chimeric PML-RARα. However, in APL cells, the SIM-containing PML from the normal allele may oligomerize with PML-RARα and facilitate RNF4-dependent degradation of the fusion protein. Furthermore, PML-RARα degradation could occur through other pathways that promote PML degradation in response to As_2_O_3_ independently of RNF4 (e.g., USP7-dependent PML degradation, see below). This may account for the delayed As_2_O_3_-induced degradation of the expressed PML-RARα in PML^−/−^ cells compared to wild-type cells (Jeanne et al., 2010; Lallemand-Breitenbach et al., 2012).

## Others Pathways of PML Degradation

In addition to the PML NB disruption that occurs upon viral infection or in APL cells, expression of the PML protein is frequently lost in human cancers from multiple origins (Gurrieri et al., 2004). Emerging studies reveal that proteasome-dependent degradation is a mechanism by which tumor cells restrict PML expression (reviewed in Chen et al., 2012). The best characterized PML degradation pathway implicates RNF4 as detailed above. Below, we will review other pathways controlling PML stability.

### Casein kinase-2

Casein Kinase-2 (CK2) is a ubiquitously expressed and highly conserved serine/threonine kinase that targets PML. After CK2-mediated phosphorylation, PML undergoes ubiquitin-mediated degradation (Scaglioni et al., 2006). Cell treatments with osmotic shock, anisomycin, or UV radiation, which result in PML phosphorylation, polyubiquitination, and degradation, may exert their effects, in part, *via* CK2. Indeed, two specific CK2 inhibitors (TBB and TBCA) abrogate osmotic shock-induced PML degradation. Scaglioni and colleagues showed that CK2 phosphorylates PMLIVa at multiple sites (S512–514 and S517) and that CK2 triggers PML degradation through the phosphorylation of PMLIVa S517 (corresponding to S565 in PMLI to PMLV) (Figures 1 and 2). These CK2-phosphorylation sites are found in the nuclear PMLI to PMLV and are missing in PMLVI as well as in PML-RARα. CK2-phosphorylation depends upon PIAS1-induced PML SUMOylation. SUMOylation enhances the interaction of PML with CK2, which leads to PML phosphorylation and degradation (Rabellino et al., 2012). Interestingly, the CK2-phosphorylation sites are not required for As_2_O_3_-induced PML degradation, since, a polyserine mutant deficient for CK2-phosphorylation (PMLIII-S560-565A) is still sensitive to As_2_O_3_-induced degradation (Percherancier et al., 2009).

**Figure 4:**
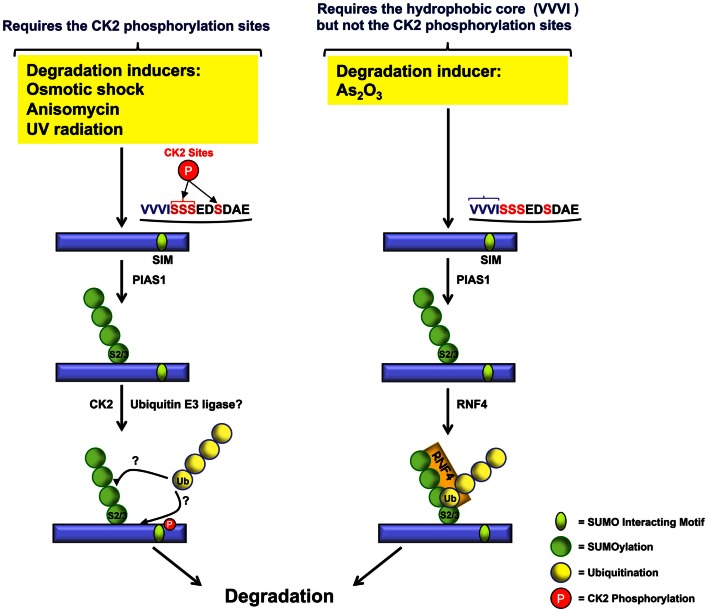
**Role of CK2 phosphorylated serines and the VVVI hydrophobic core in PML degradation**. In response to osmotic shock, anisomycin, or UV radiation, PML is phosphorylated at the CK2 serine sites (red) adjacent to the SIM hydrophobic core (VVVI) (blue) leading to PML degradation. In contrast, As_2_O_3_-induced PML degradation requires the SIM VVVI.

Therefore, as illustrated in Figure 4, different inducers use at least two independent pathways for PML degradation. As_2_O_3_-induced degradation depends upon the recruitment of RNF4 and requires the hydrophobic core of the SIM but not the CK2-phosphorylation sites. CK2-dependent PML degradation relies on CK2 sites adjacent to the hydrophobic core of the PML SIM. At present, the ubiquitin E3 ligase responsible for CK2-dependent PML degradation remains to be identified. Furthermore, it would be interesting to determine if the CK2-mediated degradation of PML triggered by various inducers such as osmotic shock, anisomycin, and UV radiation also requires the hydrophobic core of the SIM in addition to its adjacent CK2-phosphorylation sites.

Noticeably, CK2-phosphorylation sites favor the interaction of the hydrophobic core of PML SIM with SUMO-modified partners and, thus, contribute to normal PML NB function (Shen et al., 2006; Percherancier et al., 2009).

Taken together these findings suggest that CK2 and RNF4 may negatively regulate the level of PML by two independent pathways under oncogenic or physiological conditions. Interestingly PML mutants resistant to CK2-phosphorylation increase tumor suppressive functions. Also, an inverse correlation between PML protein levels and CK2 kinase activity was observed in human lung cancer-derived cell lines and primary cell cultures (Scaglioni et al., 2006, 2008).

### Arkadia

Arkadia/RNF111 is a member of the RING-finger ubiquitin ligase superfamily that promotes activation of the TGF-signaling pathway and acts as a signal-dependent E3 ubiquitin ligase for SnoN and Ski (Levy et al., 2007). Very recently, it has been reported that Arkadia is a novel poly-SUMO-targeted ubiquitin ligase which is involved in As_2_O_3_-induced PML degradation (Erker et al., 2013). Arkadia contains in its N-terminus three successive SIMs that mediate non-covalent interactions with poly-SUMO2. The third SIM, VVDL, of Arkadia is the most relevant for this interaction (Erker et al., 2013). Arkadia binds to As_2_O_3_-induced SUMO-modified PML or PML-RARα only when the SIMs of Arkadia are intact, thus indicating that Arkadia interacts with these SUMOylated substrates *via* its SIMs. Specific binding of Arkadia to poly-SUMOylated PML leads to the accumulation of Arkadia in PML NBs shortly after As_2_O_3_ cell treatment. In addition, knockdown of Arkadia (Erker et al., 2013) results, as observed for RNF4 (Tatham et al., 2008), in the accumulation of SUMOylated PML in response to As_2_O_3_. Arkadia and RNF4 do not act synergistically but most probably, act independently during this degradation process. In addition to RNF4, these new findings clearly identify Arkadia, as a second SUMO-dependent E3 ubiquitin ligase implicated in As_2_O_3_-induced degradation of poly-SUMOylated PML.

### Ubiquitin-specific protease 7

USP7/HAUSP (Herpes virus-Associated Ubiquitin-Specific Protease) was identified as an interacting partner of HSV-1 ICP0 E3 ubiquitin ligase and was found to be associated with PML NBs (Everett et al., 1997). USP7 interacts with and controls PML stability, independently of its deubiquitinase activity (Sarkari et al., 2011). CK2 and RNF4 regulators of PML catabolism are dispensable for USP7-mediated PML NB disruption and PML degradation (Sarkari et al., 2011). Thus, USP7 induces PML degradation by another pathway that does not implicate phosphorylation of PML by CK2 or polyubiquitination by RNF4. Interestingly, USP7 depletion indicates that USP7 participates in As_2_O_3_-induced degradation of PML (Sarkari et al., 2011). Since USP7 promotes PML ubiquitination, it may recruit an E3 ubiquitin ligase or an inducer of PML degradation. This represents an additional PML degradation pathway the mechanism of which remains to be determined.

### HMGA2

The High-Mobility Group A protein 2 (HMGA2) is an architectural transcription factor implicated in cell growth, regulation of transcription, and transformation. It has been reported to mediate a proteasome-dependent PML degradation. SUMOylation of HMGA2 is required to destabilize PML. In addition, As_2_O_3_ was shown to increase both HMGA2 SUMOylation and its recruitment in nuclear foci around PML NBs (Cao et al., 2008). However, the implication of HMGA2 in As_2_O_3_-induced PML degradation remains to be determined.

### Peptidyl-prolyl cis-trans isomerase

The peptidyl-prolyl cis-trans isomerase (Pin1) has been shown to bind phosphorylated PML, promoting a conformational change that results in PML degradation (Reineke et al., 2008). The interaction of Pin1 with PML is enhanced by various stimuli such as EGF or hypoxia, which induce the phosphorylation of various serine residues within exon 6 of all nuclear PML isoforms (Lim et al., 2011; Yuan et al., 2011). ERK2 and CDK1/2 are kinases involved in the phosphorylation-dependent mechanism that leads to Pin1-mediated PML degradation (Lim et al., 2011; Yuan et al., 2011). Remarkably in this case, SUMOylation of PML blocks its interaction with Pin1 and prevents its degradation (Reineke et al., 2008). This degradation process is independent of RNF4 activity and CK2-phosphorylation (Yuan et al., 2011). Interestingly, the role of the Cullin3-KLHL20 ubiquitin ligase in PML polyubiquitination and in Pin1-dependent proteasomal degradation was recently demonstrated (Yuan et al., 2011). This process requires CDK1/2 phosphorylation of PML on S518 (found in all nuclear PML and in the long PML-RARα isoform) and prolyl cis/trans isomerization of P519 by Pin1, two events which promote the interaction of KLHL20 with PML. In contrast to RNF4, KLHL20-based E3 ligase polyubiquitinates unSUMOylated PML (Yuan et al., 2011). Interestingly, higher levels of Pin1 and KLHL20 in human prostate cancer correlate with a decrease of PML expression and disease progression (Yuan et al., 2011).

### E6-associated protein

E6-Associated Protein (E6AP) was initially identified as the E3 ligase that acts with the human papillomavirus E6 protein to promote p53 degradation (Scheffner et al., 1993). E6AP is the founding member of the HECT (homologous to the E6AP C-terminus) family of ubiquitin ligases. E6AP and PML interact and colocalize in PML NBs. Also, E6AP promotes the ubiquitination and proteasomal degradation of all nuclear PML isoforms (Louria-Hayon et al., 2009; Wolyniec et al., 2012). A SUMOylation mutant, PMLIV-K160R, is still ubiquitinated and degraded by E6AP (Louria-Hayon et al., 2009). The high PML levels in multiple tissues and primary cells derived from E6AP deficient mice further demonstrate the regulation of PML by E6AP. In addition, an increase in E6AP expression is correlated with PML down-regulation in human Burkitt’s lymphoma specimens and a number of B cell lymphoma cell lines (Wolyniec et al., 2012). Inversely, down-regulation of E6AP in B cell lymphoma cells restores PML expression with a concurrent induction of cellular senescence in these cells. Taken together, these results suggest that E6AP expression contributes, in part, to PML degradation and to the loss of PML in B cell lymphoma. Also, E6AP may contribute to As_2_O_3_-induced PML degradation since exogenous E6AP, together with As_2_O_3_, markedly reduces the half-life of PML (Louria-Hayon et al., 2009).

### Herpes simplex virus 1 and Epstein–Barr virus

Studies with DNA and RNA viruses provide another example of alteration of PML NBs and regulation of PML stability. Such alterations could be a viral strategy to evade a cellular resistance mechanism. This field has been covered in numerous reviews (Everett and Chelbi-Alix, 2007; Tavalai and Stamminger, 2008; Geoffroy and Chelbi-Alix, 2011).

The best-studied virus involved in PML degradation is Herpes simplex virus 1 (HSV-1) (Everett et al., 1998; Chelbi-Alix and de The, 1999; Boutell and Everett, 2013). The immediate early protein, ICP0 protein, mediates HSV-1-induced proteasome-dependent PML degradation. This process correlates with the ability of ICP0 to interact with USP7 (Everett et al., 1997). ICP0, as a viral RING-finger ubiquitin ligase of the STUbL family, is one of the first proteins expressed during HSV-1 infection and it localizes in and disrupts PML NBs. ICP0 binds to SUMOylated PML through its own viral multiple SIM-like sequences (Boutell et al., 2011) and it induces PML degradation by two distinct mechanisms. Like RNF4, ICP0 preferentially induces the degradation of all SUMO-modified PML isoforms but, unlike RNF4, ICP0 also targets PMLI in a SUMO-independent manner (Boutell et al., 2011; Cuchet-Lourenco et al., 2012). In addition, the CK2 pathway is not implicated since CK2 inhibitors do not compromise HSV-1- or ICP0-induced PML degradation (Smith et al., 2011).

Epstein–Barr virus (EBV), another herpes virus, induces PML degradation and PML NB disruption *via* the expression of Epstein–Barr nuclear antigen 1 (EBNA1) protein. EBNA1 disrupts PML NBs by inducing the degradation of all PML isoforms (Sivachandran et al., 2008). PML degradation by EBNA1 requires two EBNA1 domains. One domain binds CK2 and the other binds USP7. These interactions result in the formation of a ternary complex between EBNA1, CK2, and USP7 (Sivachandran et al., 2008, 2010). The implication of USP7 is demonstrated by the fact that PML degradation is not observed with EBNA1 mutant defective in USP7-binding or with wild-type EBNA1 when USP7 is depleted (Sivachandran et al., 2008). Also, by using an antibody specific for PML phospho-S517, EBNA1 was shown to enhance PML phosphorylation (Sivachandran et al., 2010). Interestingly, EBV-positive gastric carcinoma tumors have reduced PML staining as compared to EBV negative samples (Sivachandran et al., 2012). Therefore, the loss of PML and PML NB disruption by EBNA1 is one mechanism by which EBV may contribute to the development of gastric cancer.

Thus, elucidating the mechanisms implicated in virus-dependent PML degradation could help to provide strategies for developing antiviral therapies that prevent viral infections.

## Intrinsic Antiviral Defense Mediated by a Specific PML Isoform

One key function of the PML NBs is to protect cells from viral infection. This antiviral defense is counteracted by viruses, which have developed various strategies using the SUMO pathway to alter the localization and/or expression of PML. A role of PML in viral resistance *in vivo* is indicated by the increased sensitivity of *PML* knockout mice (as compared to the wild-type mice) to lymphocytic choriomeningitis virus (LCMV) and to vesicular stomatitis virus (VSV) infections (Bonilla et al., 2002). These observations corroborate with *in cellulo* data which demonstrate that fibroblasts derived from these mice (PML^−/−^ MEFs) exhibit enhanced replication for RNA viruses such as LCMV (Djavani et al., 2001), rabies virus (Blondel et al., 2002), and EMCV (El Mchichi et al., 2010) (Table 3). Also, PML depletion in human cells results in enhanced replication of DNA viruses from the herpes family such as *Cytomegalovirus* (HCMV) (Tavalai et al., 2006) and VZV (Kyratsous and Silverstein, 2009) (Table 3). The implication of PML in antiviral defense against RNA and DNA viruses from different families has been demonstrated in cells depleted for PML or in cells stably expressing individual PML isoforms (reviewed in Everett and Chelbi-Alix, 2007; Geoffroy and Chelbi-Alix, 2011).

**Table 3:** **Only PMLIV confers resistance to EMCV, rabies virus, and VZV**.

Virus	Higher viral production	Inhibition of viral production	Mechanism	Reference
*RNA viruses*				
EMCV	Absence of PML *(MEFs PML^−/−^)*	PMLIV	Sequestration of 3D pol in PML NBs	El Mchichi et al. (2010); Maroui et al. (2011)
Rabies virus		PMLIV	ND	Blondel et al. (2002, 2010) *DNA virus*				
VZV	Depletion of PML *(Human cells)*	PMLIV	Sequestration of ORF23 in PML NBs	Kyratsous and Silverstein (2009); Reichelt et al. (2011)

*ND, not determined*.

Further on, we will discuss the role of PML in antiviral defense by taking examples from the picornavirus family (EMCV) (Maroui et al., 2011) and the herpes virus family (VZV) (Reichelt et al., 2011) because these studies were performed with all the PML isoforms and they demonstrate how a single PML isoform, PMLIV, confers viral resistance. Also, only PMLIV confers resistance to rabies virus by an unknown mechanism (Blondel et al., 2010) (Table 4).

**Table 4:** **EMCV and VZV alter PML localization and/or expression**.

Virus	PML NB alteration	PML degradation	Reference
EMCV	Loss of PML NBs	SUMO- and proteasome-dependent PML degradation*	El Mchichi et al. (2010)
VZV	ORF61 SIM-mediated PML NB disruption	No PML degradation in ORF61-expressing cells or in VZV-infected cells	Wang et al. (2011)

*There is no degradation of PML in infected cells stably expressing PMLIV because PMLIV inhibits EMCV production.

### PML and EMCV

Encephalomyocarditis virus belongs to the Picornaviridae family (*Cardiovirus* genus). The virion is composed of a single-stranded RNA molecule of positive polarity. Although EMCV replication occurs in the cytoplasm, it has been reported that, during the early steps of infection or in transfected cells, the viral protease 3C (3Cpro) and the 3D polymerase (3Dpol) colocalize with PML in NBs (El Mchichi et al., 2010; Maroui et al., 2011). The 3Dpol is a central element of viral RNA replication complexes and the protease 3Cpro is the central enzyme in the viral cleavage cascade.

Promyelocytic leukemia has been implicated in resistance to EMCV since PML^−/−^ MEFs are more sensitive to EMCV than wild-type cells (El Mchichi et al., 2010) and because specific PML isoforms, PMLIV, and its variant PMLIVa (missing exon 5), protect cells from EMCV infection (Table 3). PMLIV inhibits viral RNA replication by interacting with the viral polymerase, 3Dpol, and by sequestering it within PML NBs (Maroui et al., 2011). No other PML isoform is able to recruit the 3Dpol into PML NBs and to impair EMCV multiplication (Maroui et al., 2011). The SUMOylation of PML is required for its anti-EMCV activity and, accordingly, a SUMOylation-deficient mutant (PMLIV-3KR) is neither able to recruit the 3Dpol within PML NBs nor to impair viral production (Maroui et al., 2011). PMLIV and PMLIVa differ from the other PML isoforms by the presence of exons 8a and 8b in their common C-terminal region. Exon 8b of PMLIV is required for the sequestration of 3Dpol within PML NBs that leads to viral resistance. PMLIV has an intrinsic antiviral property against EMCV that occurs independently of IFN synthesis. Importantly, specific depletion of PMLIV reduces the capacity of IFN to protect cells from EMCV infection (Maroui et al., 2011). These findings reveal the mechanism by which PML confers resistance to EMCV and a new pathway for the mediation of the antiviral activity of IFN against EMCV.

Encephalomyocarditis virus has developed various strategies to counteract cellular antiviral defenses that include blocking *IFN-*α/β gene transcription (Hato et al., 2007) and degrading PML in a SUMO- and proteasome-dependent manner (El Mchichi et al., 2010) (Table 4). Remarkably, as observed with As_2_O_3_, infection with EMCV results in recruitment of PML toward the nuclear matrix followed by its degradation (Zhu et al., 1997; Porta et al., 2005; El Mchichi et al., 2010). Soon after infection, EMCV induces the transfer of PML from the nucleoplasm to the nuclear matrix, both in IFN-treated and PMLIII-expressing cells. EMCV also enhances PML conjugation to SUMO (SUMO-1, -2, and -3) which leads to an increase in PML NB size. The viral 3Cpro and the proteasome component colocalize with PML within the NBs. This process leads to PML degradation in a SUMO- and proteasome-dependent manner and to a decrease of PML NBs (Table 4). Indeed, EMCV-induced degradation requires that PMLIII bind covalently to SUMO and this degradation process is reduced in cells depleted of SUMO1 or SUMO2/3. As observed with As_2_O_3_, the SIM of PML is not required for EMCV-enhanced PML SUMOylation, but unlike As_2_O_3_ (Maroui et al., 2012), the hydrophobic core of the SIM is dispensable for EMCV-mediated PML degradation (El Mchichi et al., 2010).

As PMLIV protects cells from EMCV infection, its protein level remained constant (Maroui et al., 2011) whereas the expression level of the other nuclear PML isoforms is decreased upon EMCV infection (El Mchichi et al., 2010 and unpublished data). These results reveal a strategy used by EMCV to destabilize PML through mechanisms implicating SUMO and the proteasome. However, the SUMO-dependent pathways contributing to EMCV-induced PML degradation remain to be elucidated.

### PML and VZV

Varicella-zoster virus belongs to the Herpesviridae family and is classified as an alpha herpes virus. Two VZV proteins have important roles in viral replication. The first protein derives from an open reading frame (ORF23) that encodes a conserved capsid protein, which is specifically sequestered by PMLIV within PML NBs thus leading to VZV restriction. The second protein is ORF61 that counteracts this antiviral defense by disrupting the PML NBs.

Depletion of PML protein enhances VZV replication in cell culture, indicating a role for PML in the host cell defense (Kyratsous and Silverstein, 2009). Among the six nuclear PML isoforms (PMLI to PMLVI), only PMLIV sequesters the ORF23 capsid protein in PML NBs in infected cells and significantly inhibits viral infection (Reichelt et al., 2011) (Table 3). This antiviral property requires the unique C-terminal region of PMLIV, which contains exons 8a and 8b as the PMLIV-Δ8ab mutant is unable to interact with the ORF23 protein. Thus, the specific interaction of the C-terminal part of PMLIV with the ORF23 capsid leads to sequestration of the capsid in PML NBs and to VZV restriction. Interestingly, PML was recently shown to restrict VZV pathogenesis in human skin xenografts in mice (Wang et al., 2011).

Like other herpes viruses, VZV disrupts PML NBs for efficient replication. VZV encodes an ICP0 ortholog, open reading frame 61 (ORF61) that, similarly to ICP0, transcriptionally activates viral promoters and enhances the infectivity of viral DNA (Moriuchi et al., 1993; Mossman et al., 2000). VZV-mediated PML NB disruption is achieved by ORF61 protein *via* its SIMs. ORF61 is a viral RING-finger protein with three functional SIMs, like those present in cellular proteins such as RNF4. In the absence of functional ORF61 SIMs, PML NBs are not affected and VZV infection is impaired (Wang et al., 2011). The finding that ORF61 requires its SIMs to alter PML NBs provides new evidence that non-covalent SIM-SUMO interactions between PML and a viral protein can counteract the intrinsic anti-VZV activity mediated by PML NBs. PML protein expression is persistent in VZV-infected cells and ORF61 protein alone can cause PML NB disruption without degrading PML proteins (Table 4). Unlike RNF4, ORF61 does not promote PML degradation.

Promyelocytic leukemia NBs provide an intrinsic host defense against VZV infection and the ORF61 SIM-dependent PML NB disruption is used by VZV to counteract the antiviral activity of these nuclear structures.

## Specific Functions of the PML Isoforms

The implication of PML isoforms in various cellular processes is due to their ability to interact with different partners either in the nucleus or in the cytoplasm. Although the PML isoforms may have related functions due to their common functional RBCC/TRIM domain, increasing evidences suggest that each PML isoform possesses distinct functions mediated by its specific C-terminal sequence (Table 5). It has been reported that, in various cell lines, the endogenous expression of the PMLIII, PMLIV, and PMLV isoforms is quantitatively less as compared to PMLI and PMLII (Condemine et al., 2006).

**Table 5:** **Functions of specific PML isoforms**.

PML isoforms and exons^1^	Specific isoform functions	Reference
**PMLI (882 AA)**		
1-2-3-4-5-6-7a-8a-9	Interacts with AML1 and stimulates myeloid cell differentiation	Nguyen et al. (2005)
	Interacts with and is degraded by ICP0 in a SUMO-independent manner	Cuchet-Lourenco et al. (2012)
**PMLII (829 AA)**		
1-2-3-4-5-6-7a-7b	Implicated in Ad5 virus-induced PML NB disruption	Leppard et al. (2009)
	Interacts with Ad5 E1A-13S and enhances viral transcription	Berscheminski et al. (2013)
**PMLIII (641 AA)**		
1-2-3-4-5-6-7a-ri7ab*-7b*^2^^,^^3^	Controls centrosome duplication	Xu et al. (2005)
**PMLIV (633 AA)**		
1-2-3-4-5-6-7a**-**8a-8b	Regulates apoptosis, senescence, and DNA damage	Bischof et al. (2002); Guo et al. (2000); Pearson et al. (2000)
	Interacts with p53, PU.1, TERT, TRF1, and TIP60	Fogal et al. (2000); Oh et al. (2009); Yoshida et al. (2007); Wu et al. (2009); Yu et al. (2010)
	Destabilizes c-Myc	Buschbeck et al. (2007)
	Inhibits VZV, EMCV, and rabies virus	Maroui et al. (2011); Reichelt et al. (2011); Blondel et al. (2010)
**PMLV (611 AA)**		
1-2-3-4-5-6-7a-ri7ab^3^	Acts as a potential scaffold of PML NBs	Weidtkamp-Peters et al. (2008)
	Forms NBs and recruits Daxx and Sp100 *via* aa 591–611	Geng et al. (2012)
**PMLVI (560 AA)**		
1-2-3-4-5-6-riGTAGGGAG-7a*^4^	Resists to As_2_O_3_-induced degradation due to the lack of the SIM in exon 7a	Maroui et al. (2012)
**PMLVIIB (435 AA)**		
1-2-3-4-7b*	Activates TGFβ signaling	Lin et al. (2004)
		Carracedo et al. (2011)
**cPMLΔ5-6/PMLIb to VIb (423 aa)**		
1-2-3-4-7a*^4^^,^^5^	Sequesters ICP0 in the cytoplasm and confers resistance to HSV-1	McNally et al. (2008)

*^1^The exons encoding sequences that are specific to a single PML isoform are underlined*.

^2^Due to a frameshift, the protein sequences encoded by exon 7b* in PMLIII (VHGAHGDRRATVLASPLLASPLLASPLLASPVSAESTRSLQPALWHIPPPSLASPPAR) and 7b* in PMLVIIb (LPPPAHALTGPAQSSTH) are shorter and different from the sequence encoded by exon 7b in PMLII (259 aa).

^3^The protein sequence encoded by the retained introns ri7ab* in PMLIII (VSSSPQSEVLYWK) is different from that of the ri7ab of PMLV (VSGPEVQARTPASPHFRSQGAQPQQVTLRLALRLGNFPVRH).

^4^Due to a frameshift, the protein sequence encoded by the exon 7a* (RNALW) in PMLVI and the PMLIb to PMLVIb isoforms, which lack exons 5 and 6, is different from that of the SIM-encoding exon 7a (EERVVVISSEDSDAENS) in PMLI to PMLV.

^5^cPMLΔ5-6 corresponds to the cytoplasmic PML isoform, which lacks exons 5 and 6. Note that alternative splicing of exons 5 and 6 in the PMLI to PMLVI mRNA variants introduces a frameshift and generates proteins corresponding to PMLIb to PMLVIb, all of which have a STOP codon at the beginning of exon 7a, termed here 7a* and which code for the same cytoplasmic protein, cPMLΔ5-6, corresponding to PMLIb to PMLVIb in the Jensen nomenclature.

*ri, retained intron*.

### Nuclear PML isoforms

Nuclear PML is considered to be the organizer protein of the PML NBs. All six human nuclear PML isoforms are able to form NBs when expressed in PML-negative cells (Brand et al., 2010). All nuclear PML isoforms (PMLI to PMLVI), but not the cytoplasmic one (PMLVIIb), function as a positive regulator of IFNγ signaling and this process requires PML SUMOylation (El Bougrini et al., 2011). The functions of the nuclear PML isoforms have been studied mainly in the context of their localization within the NBs where PML is concentrated. To date, their biological functions in the nucleoplasm remains largely unexplored despites the fact that the majority of PML expressed in the cell is dispersed diffusely in the nucleoplasm.

PMLI interacts with acute myeloid leukemia 1 (AML1) to induce differentiation of the hematopoietic cell lineage (Nguyen et al., 2005). Also, PMLI interacts and colocalizes with HSV-1 ICP0 owing to its specific C-terminus sequence encoded by exon 9. This leads to SUMO-independent degradation of PMLI by ICP0 (Boutell et al., 2011; Cuchet-Lourenco et al., 2012). To the contrary, the other nuclear PML isoforms are degraded by ICP0 using a SUMO-dependent mechanism.

PMLII binds specifically to adenovirus type 5 (Ad5) E4 Orf3 *via* its C-terminus. This interaction, which is conferred by 40 amino acid residues encoded by PMLII exon 7b (amino acids 645–684), mediates the virus-induced PML NB disruption (Leppard et al., 2009). Recently, it has been reported that PMLII also physically interacts with Ad5 E1A–13S to positively activate viral transcription (Berscheminski et al., 2013). This suggests that, in contrast to PML NB-associated antiviral defense, PMLII may assist Ad5 viral transcription.

PMLIII was shown (using PML isoform-specific antibodies) to play a direct role in the control of centrosome duplication through suppression of Aurora A activation, which prevents centrosome reduplication (Xu et al., 2005).

PMLIV is the most studied PML isoform. PMLIV specifically interacts with many cellular proteins such as p53, PU.1, TERT (telomerase reverse transcriptase), TRF1 (telomeric repeat binding factor 1), and the histone acetyl transferase TIP60 (Fogal et al., 2000; Yoshida et al., 2007; Oh et al., 2009; Wu et al., 2009; Yu et al., 2010). PMLIV recruits and activates p53 in PML NBs and enhances apoptosis and senescence following various stimuli (Guo et al., 2000; Pearson et al., 2000; Bischof et al., 2002; Bernardi et al., 2004). Although several PML isoforms are able to interact with c-Myc, the ability to destabilize c-Myc is specific to PMLIV (Buschbeck et al., 2007). In addition, studies performed with all PML isoforms demonstrate that only PMLIV protects cells from infection with rabies virus, VZV, or EMCV (Blondel et al., 2010; Maroui et al., 2011; Reichelt et al., 2011). This demonstrates the importance of exons 8a and 8b in mediating protein interactions and, thus, specific functions.

PMLV was identified as a potential scaffold for the PML NB since comparative kinetic analyses with various GFP-tagged PML isoforms reveal that PMLV exhibits the longest residence time (Weidtkamp-Peters et al., 2008). This suggests that specific C-terminus of PMLV, which harbors the translated intron 7ab, may contribute to PML NB structural stability. Also, the 21-amino acid-long region within PMLV (amino acids 591–611) is sufficient for NB formation and the recruitment of PML NB partners including Daxx and Sp100. This process occurs independently of the N-terminal RBCC domain and endogenous PML (Geng et al., 2012).

PMLVI is the shortest nuclear isoform encoded only by exons 1–6 due to a STOP codon located at the beginning of exon 7a. Therefore it does not contain the SIM hydrophobic core and the CK2-phosphorylation sites. As compared to all other nuclear isoforms, PMLVI was shown to be resistant to As_2_O_3_-induced PML degradation due to the lack of the SIM hydrophobic core (Maroui et al., 2012).

### Cytoplasmic PML isoforms

Although the majority of PML proteins are in the nucleus, nucleo-cytoplasmic fractionation reveals that a fraction of PML is found in the cytoplasm (Lin et al., 2004; Giorgi et al., 2010). Emerging data suggest the implication of cytoplasmic PML in cytokine signaling (Lin et al., 2004), apoptosis (Giorgi et al., 2010), and antiviral defense (McNally et al., 2008).

Alternative splicing can generate various cytoplasmic PML isoforms. PMLVIIb, which lacks exons 5 and 6, is cytoplasmic (Jensen et al., 2001). Also, an identical cytoplasmic PML protein, cPMLΔ5-6, can be generated from all PML mRNA variants that lack exons 5 and 6 (corresponding to PMLIb to PMLVIb as mentioned above in Section Nomenclature of PML Isoforms).

Furthermore, different cytoplasmic PML proteins with their preserved specific C-terminal sequences can be generated, potentially, from PML mRNA variants missing exons 4, 5, and 6 (PMLIc to PMLVIc). However, further investigations are needed to demonstrate that all these cytoplasmic isoforms, indeed, are expressed endogenously.

The first evidence, revealing a cytoplasmic function for PML, was obtained for PMLVIIb or cPML, where cPML is “cPML3 3-7” missing exons 4-5-6 that corresponds to PMLIVc in the Jensen nomenclature (Lin et al., 2004; Carracedo et al., 2011). This study showed that cytoplasmic PML is essential for the activation of transforming growth factor beta (TGFβ) signaling through its interaction with SMAD2/3 and Smad anchor for receptor activation (SARA).

Another study showed that HSV-1 infection leads to an alteration of PML pre-mRNA splicing which leads to the elimination of exons 5 and 6 and the insertion of a STOP codon at the beginning of exon 7a. The resulting translation product is cPMLΔ5-6. The expression of cPMLΔ5-6 confers resistance to HSV-1 by sequestering the immediate early HSV-1 protein ICP0 in the cytoplasm (McNally et al., 2008). The anti-HSV-1 effect of cPMLΔ5-6 is higher in wild-type MEFs as compared to PML^−/−^ MEFs which suggests the participation of other PML isoforms (McNally et al., 2008). These findings provide the first example of a protective effect of cytoplasmic PML against viral infection.

## Concluding Remarks

In this review, we have discussed the nomenclature and structural organization of PML isoforms in order to clarify the various designations and classifications found in different databases. We also have summarized the functions mediated by the specific C-terminal end of the different PML isoforms. However, much remains to be discovered about the role of the PML isoforms in the different cellular compartments, i.e., the cytoplasm, the nucleoplasm, and the nuclear matrix-associated NBs.

The different names given to identical PML isoforms in the different classification systems often make PML nomenclature confusing in publications. Therefore, it is important to include the accession numbers in future publications for the proper identification of the specific PML isoform(s) being studied.

Additional studies performed with all the PML isoforms undoubtedly will clarify further the relationships between specific functions and specific isoforms. Also, it will be of interest to determine whether or to what extent a function attributed to one isoform is maintained in the absence of the other isoforms. Consequently, the analysis of specific PML isoforms may reveal novel interacting partners and functions.

Recently, it has been reported that, contrary to PML NB-associated antiviral defense, PMLII interacts with Ad5 E1A-13S to positively activate viral transcription. Further studies will determine whether other viral proteins could take direct advantage of PML to increase viral production.

In addition to its role in the nucleus, PML has been found to accumulate in the cytoplasm and to play a critical role in cytokine signaling, antiviral response, and apoptosis. This demonstrates that PML also exerts specific functions in this compartment. Further research is needed to confirm which of the cytoplasmic PML isoforms expected from alternative splicing are, indeed endogenously expressed and whether post-translational modifications regulate their functions.

Diverse cellular stimuli induce proteasome-dependent PML degradation through alternative or potentially complementary pathways, some of which depend upon PML SUMOylation. Multiple cellular pathways for degradation of PML have emerged at present. They use as key players CK2, USP7, Pin1, and the three E3 ubiquitin ligases, E6AP, Arkadia, and RNF4. The best understood pathway, at present, is the RNF4-dependent pathway. Further investigations are required to unravel the respective involvement of E6AP, RNF4, and Arkadia in PML degradation in response to As_2_O_3_. Whereas RNF4 and Arkadia require PML SUMOylation, the Cullin3-KLHL20 ubiquitin ligase, acting in the Pin1 pathway, and E6AP can ubiquitinate unSUMOylated PML. The E3 ubiquitin ligases involved in the CK2 and USP7 PML degradation pathways are still unknown. Whether these different pathways cooperate under certain physiological or pathological conditions remains to be elucidated.

The implication of RNF4 in PML degradation in response to As_2_O_3_ is well demonstrated. Interestingly, while this review was under revision, it was shown that RNF4 ubiquitinates poly-SUMOylated Nrf2, leading to degradation of the modified Nrf2 in PML NBs in a proteasome-dependent manner (Malloy et al., 2013). Whether RNF4 can induce degradation of other SUMOylated and NB-associated proteins is unknown. As the overexpression of RNF4 alone is sufficient to induce a proteasome-dependent PML degradation (Percherancier et al., 2009), it will be interesting to assess whether RNF4 expression is increased in cancer and whether this can be correlated with a decrease of PML expression and tumor aggressiveness.

## Conflict of Interest Statement

The authors declare that the research was conducted in the absence of any commercial or financial relationships that could be construed as a potential conflict of interest.
